# Effects of Four Compounds from* Gentianella acuta* (Michx.) Hulten on Hydrogen Peroxide-Induced Injury in H9c2 Cells

**DOI:** 10.1155/2019/2692970

**Published:** 2019-01-20

**Authors:** Kai Ren, He Su, Li-juan Lv, Le-tai Yi, Xue Gong, Lian-sheng Dang, Rui-fen Zhang, Min-hui Li

**Affiliations:** ^1^Baotou Medical College, Baotou 014000, China; ^2^Inner Mongolia Autonomous Region Hospital of Traditional Chinese Medicine, Hohhot 010020, China; ^3^Inner Mongolia Institute of Traditional Chinese Medicine, Hohhot 010020, China; ^4^Department of Basic Science, Tianjin Agricultural University, Tianjin 300384, China; ^5^Department of Geriatrics, The first Affiliated Hospital of Baotou Medical College, Baotou 014000, China

## Abstract

In previous studies,* Gentianella acuta* (Michx.) Hulten was reported to contain xanthones, iridoids, terpenoids, and sterols and is mainly used to cure hepatitis, jaundice, fever, headache, and angina pectoris. In this study, we used bioassay guided fractionation to identify compounds from* G. acuta* and investigated their activity against hydrogen peroxide (H_2_O_2_)-induced apoptosis of H9c2 cells using the 3-(4,5-dimethylthiazol-2-yl)-2,5-diphenyltetrazolium bromide (MTT) method. The levels of nuclear factor erythroid 2-related factor 2 (Nrf2), heme oxygenase-1 (HO-1), and glutamate-cysteine ligase catalytic (GCLC) expression were assessed using quantitative real-time polymerase chain reaction (qRT-PCR). Protein expression was evaluated using western blot. The results showed that all four compounds had protective effects on H9c2 cells. The transcription levels of HO-1 and GCLC significantly increased in H9c2 cells pretreated with norswertianolin (**1**), swetrianolin (**2**), demethylbellidifolin (**3**), and bellidifolin (**4**). However, compared to the model group, the transcription levels of Nrf2 were not enhanced by pretreatment with compounds** 1**,** 2**, and** 4**. The protein expression levels of HO-1 and GCLC in H9c2 cells were greater than that in the H_2_O_2_-treated group, and the expression of Nrf2 was not significantly changed except by swetrianolin treatment; inhibitors can reverse the protective effect by ZnPP (15 *μ*M), BSO (10 *μ*M), and brusatol (10 *μ*M). The results indicated that the four compounds isolated from* G. acuta* inhibited the oxidative injury induced by H_2_O_2_ by activating the Nrf2/ARE pathway in H9c2 cells and provide evidence that* G. acuta* may be a potential therapeutic agent for the treatment of cardiovascular diseases.

## 1. Introduction

*Gentianella acuta* belongs to the family Gentianaceae and is also known by the Mongolian name Agute-Qiqige. It is an annual herbaceous plant, widely distributed to the north of China, the Mongolia Plateau, Siberia, and the Far East areas of Russia [1]. In previous studies,* G. acuta* was reported to contain xanthenes, iridoids, terpenoids, and sterols [2]. The whole grass is used as a Mongolian native medicine, mainly to treat hepatitis, jaundice, fever, and headache. A study showed that* G. acuta* had a significant effect on angina pectoris and other diseases [3].

Angina pectoris, the most common type of coronary heart disease, is a clinical syndrome caused by coronary insufficiency and acute and temporary myocardial ischemia [4]. Myocardial ischemia and reperfusion produce numerous reactive oxygen species (ROS) that can lead to oxidative injury of H9c2 cells, which is one of the main causes of H9c2 cells death after angina [5, 6]. Hydrogen peroxide (H_2_O_2_) is an important source of ROS, which directly oxidize lipids and proteins on the cell membrane, which then react with Fe^2+^ to produce ·OH and other more active free radicals to induce apoptosis or necrosis. H_2_O_2_ is one of the important causes of myocardial apoptosis through the induction of oxidative stress [7].

In this study, we used a bioassay guided method to isolate four compounds from* G. acuta*. We used chromatography to separate these active ingredients by analyzing the activity, discarding the inactive components, and repeating this process until the active ingredients were identified. We described the isolation and identification of these compounds, along with the evaluation of their inhibitory effects on H9c2 cells. To this end, we used a combination of high-performance liquid chromatography (HPLC), the 3-(4,5-dimethylthiazol-2-yl)-2,5-diphenyltetrazolium bromide (MTT), and quantitative real-time polymerase chain reaction (qRT-PCR) methods to investigate the protective effects and preliminary mechanism of action of four compounds isolated from* G. acuta*. Furthermore, the effects on protein expression of relevant pathway molecules were evaluated using western blotting.

## 2. Materials and Methods

### 2.1. Materials

The rat embryonic ventricular myocardial cell line (H9c2) was purchased from Shanghai Ruchu Biotechnology Co. Ltd. High glucose DMEM (Gibco, USA), fetal bovine serum, penicillin and streptomycin (HyClone, Thermo Scientific), phosphate-buffered saline (PBS), dimethyl sulfoxide (DMSO) purchased from the Sino-American Biotechnology Company of Beijing (Beijing, China), ultrapure water made by Gen Pure (Thermo, USA), ethanol, ethyl acetate,* n*-butanol, petroleum, chromatographic grade acetonitrile, and all chemicals were purchased from Concord Technology Co. Ltd. (Tianjin, China). H_2_O_2_ was from Tianjin Fu Yu Reagent Co., Ltd. (Tianjin, China). Trizol was purchased from Invitrogen (Carlsbad, CA, USA). PrimeScript™ RT Reagent Kit (Perfect Real Time) was obtained from TaKaRa Biotechnology Co. (Dalian, China). The primers of HO-1, GCLC, Nrf2, and GAPDH were synthesized from Sangon Biotech (Shanghai, China).

### 2.2. Plant Materials

*G. acuta* was collected from Genhe City, Inner Mongolia Autonomous Region, in July 2013 and identified by Dr. Zhang Chunhong (Baotou Medical College). A voucher specimen was deposited at the Laboratory of Pharmacognosy and Phytochemistry in Baotou Medical College.

### 2.3. Extraction and Isolation

#### 2.3.1. Preparation of Crude Extracts

Air-dried powder of* G. acuta* (301 g) was extracted successively with 70% ethanol to produce 99 g of the extract. The extract was dissolved in water and then extracted successively with petroleum ether, ethyl acetate, and *n*-butanol to produce 5.94 g, 15.37 g, and 13.61 g of dry extracts, respectively. The aqueous phase contained 15.52 g of the residue.

#### 2.3.2. Preparation of Fractions from Crude Extracts

The ethyl acetate fraction (10 g) was separated using a dry column with silica gel (200-300 mesh [150 g] and a petroleum ether-ethyl acetate mixture,7:3 v/v) to produce four main fractions (Frs. A1-A4). Fr. A2 (6.91 g) was then further purified using chromatography with silica gel (200-300 mesh, 150 g; petroleum ether-ethyl acetate,7:3 v/v) to produce 14 fractions (Frs. B1-B14). We then divided the products into three fractions (B1 [1.4 g], B2-B10 [4.47 g], and B11-B14 [0.86 g]) using thin-layer chromatography (TLC).

The *n*-butanol fraction (10 g) was chromatographed using a silica gel column (200-300 mesh, 200 g) to obtain four fractions (Frs. C1-4 [1.72, 5.7, 1.53, and 0.48 g, respectively]).

#### 2.3.3. Preparation of Monomeric Compounds

Fractions B2-B10 were further purified repeatedly using a Sephadex LH-20 column (with petroleum ether-ethyl acetate, 7:3 v/v) to obtain compounds** 1** (22 mg) and** 2** (18 mg).

Purification of Fr. C2 from the *n*-butanol fraction using repeated Sephadex LH-20 column chromatography (Sephadex LH-20, 8 cm × 80 cm) eluted with 60% methyl alcohol provided compounds** 3** (21 mg) and** 4 **(15 mg).

### 2.4. Identification of Antioxidant Compounds

#### 2.4.1. Cell Culture and Treatment

The H9c2 cells were cultured in high glucose Dulbecco's modified Eagle's medium (DMEM) supplemented with 10% fetal bovine serum (FBS) and a mixture of penicillin and streptomycin. The cells were maintained in a humidified incubator with an atmosphere of 5% CO_2_ at 37°C.

#### 2.4.2. Determination of H_2_O_2_ Model

We determined the appropriate concentration of H_2_O_2_ using real-time cellular analysis (RTCA). H9c2 cells were seeded in a CIM-plate-16 microplate in a volume of 100 *μ*L and incubated for 12 h at 37°C in an atmosphere of 5% CO_2_. The H9c2 cells were divided into control and model groups. Then, 10 *μ*L H_2_O_2_ (100, 200, 400, 600, and 800 *μ*mol·L^−1^) was added to the model groups, and the control groups were treated with 10 *μ*L DMEM complete medium, set up in three complex wells. The E-Plate 16 was replaced in the xCELLigence RTCA, monitored every 15 min, and recorded for 30 h. The effect of H_2_O_2_ on the H9c2 cells was analyzed using the xCELLigence RTCA data analysis software. The cell CI value was used to determine the optimal H_2_O_2_ concentration and duration of action.

#### 2.4.3. Cell Viability Assay

H9c2 cells were seeded in 96-well plates at a density of 2 × 10^4^ cells/well at a final volume of 100 *µ*L, and then they were divided into three groups: control, model, and treatment groups. Then, the treatment groups were treated with various concentrations of the crude extracts (0.4, 0.2, 0.1, 0.05, and 0.025 mg* ∙* mL^−1^) and the four monomeric compounds (50, 25, 12.5, 6.25, and 3.125 *μ*M) at five concentrations. After pretreatment for 12 h, 400 *μ*M H_2_O_2_ solution was added to each well, followed by 10 *µ*L MTT solution for 4 h at 37°C, and then the supernatant was discarded and 150 *µ*L dimethyl sulfoxide (DMSO) was added to each well. The plates were oscillated at a low speed for 5 min at room temperature until all the crystals that had formed were fully dissolved. The optical density was determined at an absorbance wavelength of 570 nm.

H9c2 cells were preincubated with zinc protoporphyrin IX (ZnPP, 15 *μ*M), L-buthionine-sulfoximine (BSO, 10 *μ*M), and Brusatol (10 *μ*M) for 30 min, then four compounds (12.5 *μ*M) were added for 12 h, and the cells were incubated with 400 *μ*M H_2_O_2_. After treatment, the survival cells were determined by MTT assay.

### 2.5. HPLC Detection

The following chromatographic conditions were used: Waters Symmetry C18 column (4.6 mm × 150 mm, 5 *μ*m); mobile phase: acetonitrile-0.1% phosphoric acid water; flow rate, 1.0 mL* ∙* min^−1^; detection wavelength: 254 nm; injection volume, 10 *μ*L; and gradient elution, 55 min (Table 1) [8, 9].

### 2.6. qRT-PCR

The gene expression levels of HO-1, GCLC, and Nrf2 were assessed using qRT-PCR. Briefly, total RNA was prepared from cultured H9c2 cells using TRIzol reagent and reverse-transcribed using the PrimeScript RT Master kit according to the manufacturer's instructions [10]. Aliquots of the obtained cDNA samples were then amplified using PCR with the following schedule: 40 PCR cycles at 95°C for 5 s, primer annealing at 60°C for 34 s, and extension at 72°C for 35 s. All primers were tested, the fluorescent signals were recorded, the △Ct values were calculated, and the relative values were compared to control group. The following equation was used: △Ct = Ct (sample)-Ct (endogenous control), △△Ct = △Ct (sample)-△Ct (untreated), and fold change = 2^-△△Ct^. Glyceraldehyde-3-phosphate dehydrogenase (GAPDH) was used as an internal control. The sequences of the forward/reverse PCR primers are as follows: HO-1: forward 5′-GCCTGCTAGCCTGGTTCAAG-3′, reverse 5′-AGCGGT GTCTGGGATGAACTA-3′; GCLC: forward 5′-GTCCTCAGGTGACATTCCAAG C-3′, reverse 5′-TGTTCTTCAGGGGCTCCAGTC-3′; Nrf2: forward 5′-TTGGCAG AGACATTCCCATTTG-3′, reverse 5′-AAACTTGCTCCATGTCCTGCTCTA-3′; GAPDH: forward 5′-AAGCTGGTCATCAACGGGAAAC-3′, reverse 5′-GAAGACG CCAGTAGACTCCACG-3′.

### 2.7. Western Blotting

The cells were washed with phosphate-buffered saline (PBS) and lysed on ice using radioimmunoprecipitation assay (RIPA, Beyotime, Jiangsu, China). The protein concentration was determined using a bicinchoninic acid (BCA) protein assay kit according to the manufacturer's instructions [11]. The samples were boiled at 98°C for 3 min, separated using 12% sodium dodecyl sulfate (SDS)-polyacrylamide gel electrophoresis (PAGE), and then the proteins were transferred onto polyvinylidene fluoride (PVDF) membranes, which were blocked with 5% non-fat dried milk for 60 min at room temperature. The blots were then incubated with the following rabbit polyclonal antibodies: anti-GAPDH (1:10000), anti-HO-1 (1:1000), anti-GCLC (1:1000), and anti-Nrf2 (1:1000). The blots were then washed extensively and incubated with horseradish peroxidase (HRP)-conjugated affinity pure goat anti-rabbit IgG (H+L, 1:5000). Visualization was performed using an enhanced chemiluminescence kit according to the instructions of the manufacturer.

### 2.8. Statistical Analysis

The data were analyzed using the statistical package for the social sciences (SPSS) 19.0, and the results are presented as the means ± standard deviation (SD) of three independent experiments.* P*-values < 0.05 were considered statistically significant.

## 3. Results

### 3.1. Establishment of H_2_O_2_ Model

**Figure 1:**
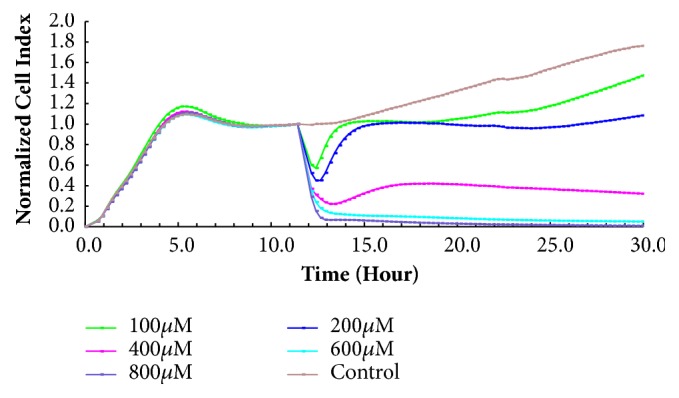
Establishment of H_2_O_2_ model.

As shown in Figure 1, 600 and 800 *μ*mol·L^−1^ H_2_O_2_ were too high to cause the appropriate level of cell death, whereas 200 *μ*mol·L^−1^ had little effects on the cells. Therefore, we determined the appropriate concentration of H_2_O_2_ to be 400 *μ*M for mimicking oxidative stress-induced injury to H9c2 cells.

### 3.2. Crude Extracts Prevented H_2_O_2_-Induced Reduction in Cell Viability

**Figure 2:**
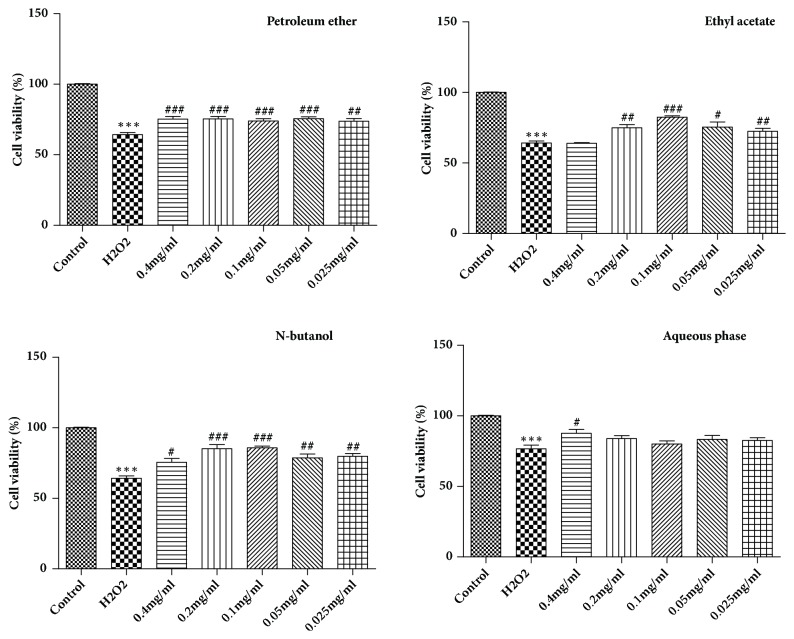
The activity detection of four crude extracts protected H9c2 cells from H_2_O_2_-induced apoptosis (means ± SD, n = 3). *∗∗∗P* < 0.001 compared with control; ^###^*P* < 0.001, ^##^*P* < 0.01, ^#^*P* < 0.05 compared with H_2_O_2_.

We used an MTT assay to analyze the effects of four crude extracts on H9c2 cells. As shown in Figure 2, the number of apoptotic cells significantly increased in the model group compared to the treatment group. Pretreatment with the four crude extracts for 12 h showed that the ethyl acetate fractions and *n*-butanol extract significantly increased the percentage of viable H9c2 cells, suggesting that the two fractions protected H9c2 cells from apoptosis induced by H_2_O_2_.

**Figure 3:**
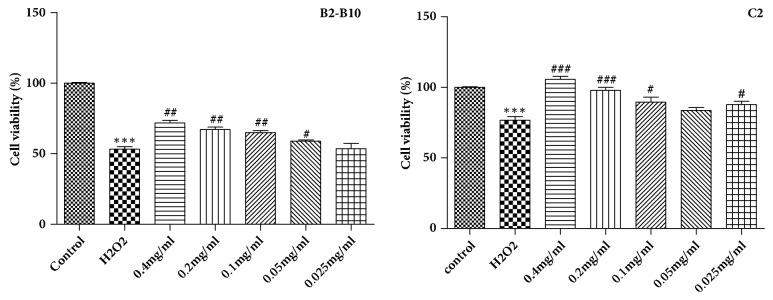
The activity detection of fractions B2-B10 and C2 on H_2_O_2_-induced apoptosis on H9c2 cells (means ± SD, n = 3). *∗∗∗P* < 0.001 compared with control; ^###^*P* < 0.001, ^##^*P* < 0.01, ^#^*P* < 0.05 compared with H_2_O_2_.

Based on these results, we further evaluated the activity of seven subfractions (B1, B2-B10, B11-B14, C1, C2, C3, and C4) from the ethyl acetate and* n*-butanol fractions. The results show that B2-B10 and C2 significantly inhibited H_2_O_2_-induced apoptosis of H9c2 cells. The protective effects of B2-B10 and C2 are shown in Figure 3 and the results of inactive fractions are not shown in the manuscript.

**Figure 4:**
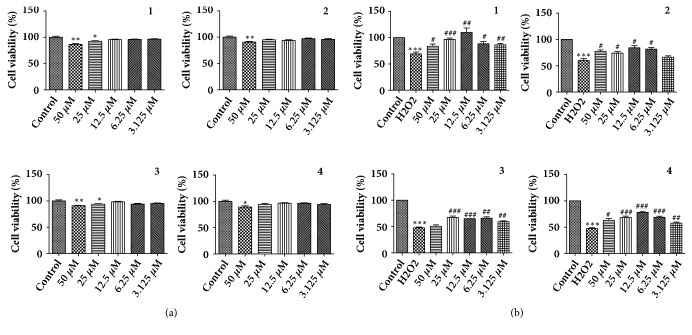
The activity detection of compounds 1, 2, 3 and 4 on H_2_O_2_-induced apoptosis on H9c2 cells. (a) Toxicity of four compounds on H9c2 cells. (b) The active ingredients on H9c2 cells assessed by MTT. (means ± SD, n = 3). *∗∗∗P* < 0.001, *∗∗P* < 0.01, *∗P* < 0.05 compared with control; ^###^*P* < 0.001, ^##^*P* < 0.01, ^#^*P* < 0.05 compared with H_2_O_2_.

We further evaluated the activity of compounds** 1**,** 2**,** 3**, and** 4** obtained from the fractions B2-B10 and C2. These results indicated that the four compounds showed no significant toxic effect on H9c2 cells. Compared with the control group, H_2_O_2_ model group could significantly reduce the cell viability, indicating that the H_2_O_2_ model is successful. The results showed that the monomeric compounds** 1**,** 2**,** 3**, and** 4** had the protective effects on H_2_O_2_-induced H9c2 cells (Figure 4).

### 3.3. HPLC Detection

**Figure 5:**
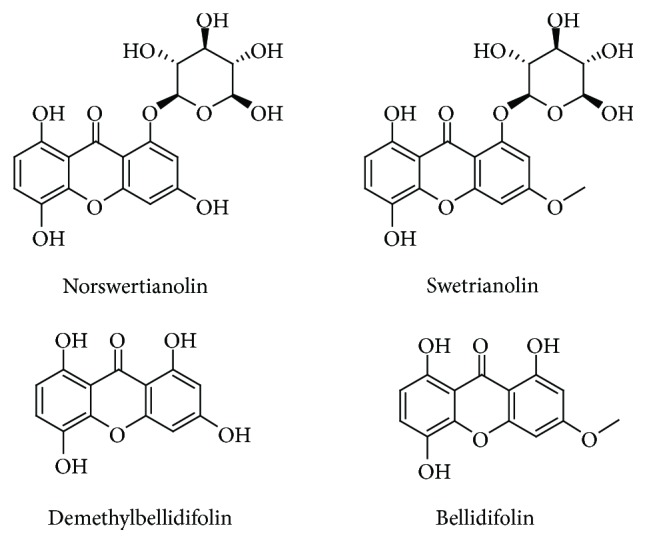
The chemical structures of four monomeric compounds.

**Figure 6:**
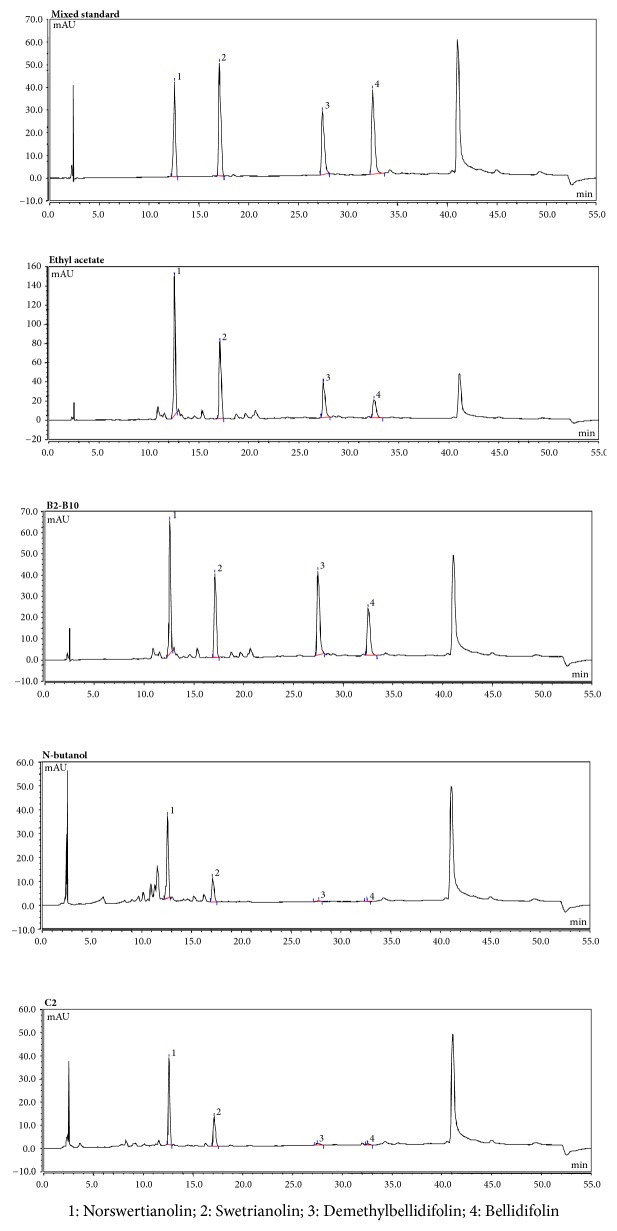
The fingerprints of the four compounds in different extracts.

The structures of these compounds were identified by comparing spectral data (proton nuclear magnetic resonance [^1^H-NMR], ^13^C-NMR, and electrospray ionization [ESI-MS]) with published data as norswertianolin (**1**), swetrianolin (**2**), demethylbellidifolin (**3**), and bellidifolin (**4**). The chemical structures of the four compounds are shown in Figure 5. We compared the plant contents of the four monomeric compounds using the area normalization method. These data indicated that there was a significant variation in the contents of the four monomeric compounds. The fingerprints of the four compounds and crude fractions are shown in Figure 6 and the relative contents are shown in Table 2.

### 3.4. Transcription Levels of HO-1, GCLC, and Nrf2

**Figure 7:**
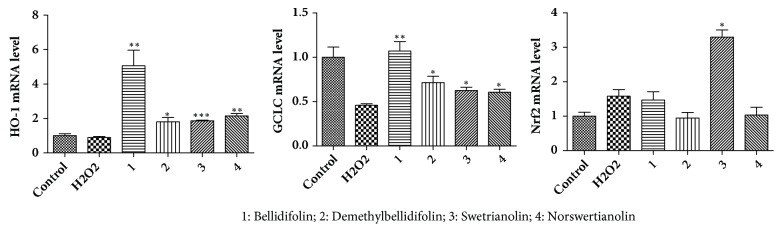
Analysis of HO-1, GCLC and Nrf2 mRNA on H9c2 cells (means ± SD, n = 3). ^*∗∗∗*^*P* < 0.001, ^*∗∗*^*P* < 0.01, ^*∗*^*P* < 0.05 compared with H_2_O_2_.

We examined the transcription levels of HO-1, GCLC, and Nrf2 in H9c2 cells treated with compounds** 1**,** 2**,** 3**, and** 4**. As shown in Figure 7, the transcription levels of HO-1 and GCLC significantly increased in H9c2 cells pretreated with the indicated concentrations of compounds** 1**,** 2**,** 3**, and** 4** for 12 h. Furthermore, the transcription levels of Nrf2 were increased in H9c2 cells pretreated with compound** 3**, whereas compounds** 1**,** 2**, and** 4** did not alter the express compared with the model group.

### 3.5. Western Blot Analysis of HO-1, GCLC, and Nrf2

**Figure 8:**
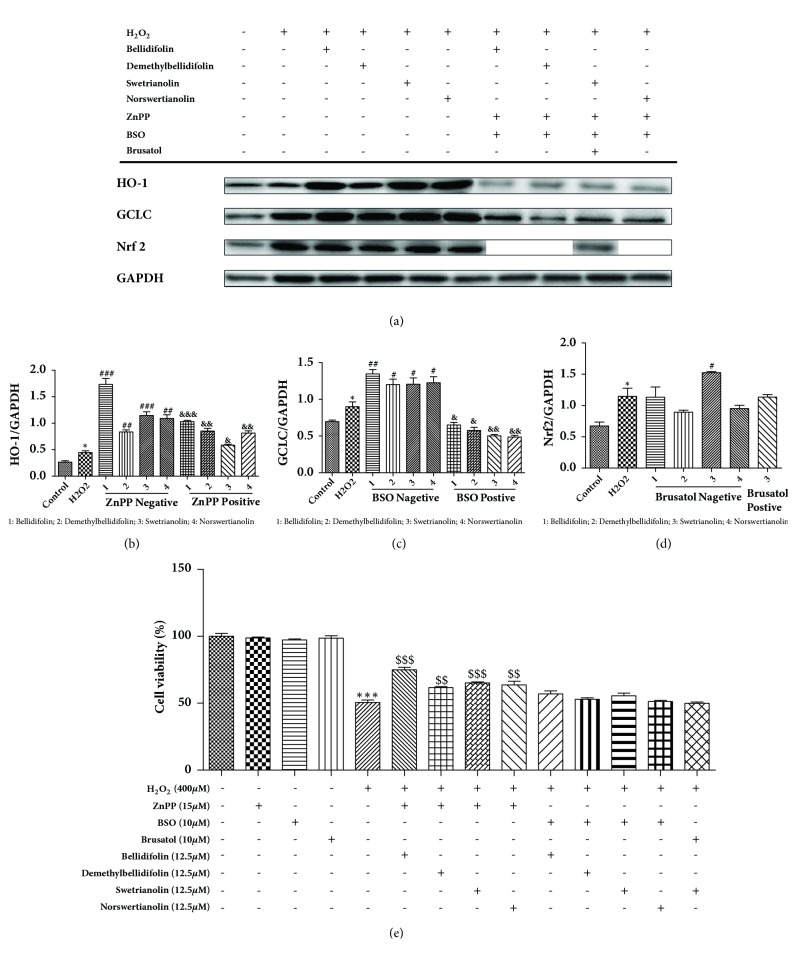
Analysis of HO-1, GCLC and Nrf2 protein levels on H9c2 cells. (a) Western blotting analysis HO-1, GCLC, Nrf2 protein expression with or without ZnPP, BSO, and brusatol (HO-1group only added ZnPP, GCLC group only added BSO, Nrf2 group only added brusatol). (b) Western blot analysis HO-1 protein expression with or without ZnPP. (c) Western blot analysis GCLC protein expression with or without BSO. (d) Western blot analysis Nrf2 protein expression with or without brusatol. (e) Effect of the different treatments on H9c2 cells. Data are expressed as the means ± SD, n = 3. ^*∗∗∗*^*P* < 0.001, ^*∗*^*P* < 0.05 compared with control group. ^###^*P *< 0.001, ^##^*P *< 0.01, ^#^*P* < 0.05 compared with H_2_O_2_ group. ^$$$^*P* < 0.001, ^$$^*P* < 0.01 compared with H_2_O_2_ group combined with four compounds. ^&&&^*P *< 0.001, ^&&^*P *< 0.01, ^&^*P* < 0.05 compared with H_2_O_2_ group combined with inhibitors and four compounds. GAPDH was used as an internal control.

Figure 8 shows the expression of HO-1 and GCLC protein levels in H9c2 cells after treatment with 400 *μ*M H_2_O_2_, and the fluorescent intensities of HO-1 and GCLC in the treatment groups were greater than that in the H_2_O_2_-treated group (Figures 8(b) and 8(c)). However, none of the compounds except for swetrianolin significantly changed the expression of Nrf2 (Figure 8(d)). Pretreatment of H9c2 cells with ZnPP, BSO, and brusatol for 30 min and then addition of four compounds for 12 h, followed by exposure to H_2_O_2_, markedly decreased the cell viability, indicating that inhibitors can reverse the protective effects of four compounds (Figure 8(e)).

## 4. Discussion

In this study, we established a bioassay-guided method to detect the antioxidative activity of* G. acuta* and found that H_2_O_2_ exposure significantly activated oxidative stress in H9c2 cells. This method can also be used to guide the more rapid and accurate separation of active ingredients than that achieved using conventional separation methods, which facilitates elucidation of the biological activity of medicinal plants, and is more conducive to the discovery of active or lead compounds. Therefore, the bioassay guided model combined with cell activity evaluation has important applicability in natural product research.

Numerous studies have shown that the Nrf2/ARE pathway is critical to several antioxidant systems [12, 13]. The transcription factor Nrf2 regulates the oxidative stress response in cells [14, 15]. Nrf2 combines with Keap1 stably under normal physiological conditions. However, following activation, Nrf2 affects the expression of downstream antioxidant proteins such as GCLC and HO-1 to enhance the antioxidant capacity of the cells. Therefore, we choose the Nrf2/ARE pathway to elucidate the underlying protective mechanism in H9c2 cells.

We established a method to separate and analyze compounds** 1**,** 2**,** 3**, and** 4 **from* G. acuta* and conducted studies to validate the efficiency of the method. This specific model achieved good separation and the four compounds were successfully eluted from the* G. acuta *sample. The crude extract had protective effect on H9c2 cells from H_2_O_2_-induced apoptosis. The activity of the monomeric compounds obtained from the fractions was then tested. Results of the cytoprotective study showed that the chemical components in* G. acuta* markedly increased cell viability compared to H_2_O_2_ alone.

The transcription levels of HO-1 and GCLC significantly increased in H9c2 cells pretreated with compounds** 1**,** 2**,** 3**, and** 4**. However, compared to the model group, the expression of HO-1 and GCLC protein levels in H9c2 cells increased, whereas that of Nrf2 was not significantly changed except with swetrianolin. Finally, our data demonstrate that* G. acuta* protects H9c2 cells from oxidative damage via the Nrf2-ARE pathway.

In conclusion, the results of the present study demonstrated that four compounds are able to protect H9c2 cells from H_2_O_2_-induced damage, which may be associated with the activation of Nrf2/ARE pathway in H9c2 cells. These results provide evidence that* G. acuta* may be a potential therapeutic agent for the treatment of cardiovascular diseases.

## Figures and Tables

**Table 1:** The chromatographic conditions.

Time (min)	Flow (mL *∙* min^−1^)	Acetonitrile (%)	0.1% phosphoric acid water (%)
0	1.0	15	85
5	1.0	15	85
10	1.0	20	80
20	1.0	30	70
40	1.0	65	35
50	1.0	65	35
50.1	1.0	15	85
55	1.0	15	85

**Table 2:** Relative content of four monomeric compounds in different extracts.

fraction	compounds			
	Relative content/%			
	1 (%)	2 (%)	3 (%)	4 (%)
ethyl acetate crude fraction	26.43	19.85	11.18	7.22
B2-B10 fraction	11.61	10.04	12.43	8.03
*n*-butanol crude fraction	6.81	2.60	0.17	0.03
C2 fraction	6.53	3.23	0.31	0.14

## Data Availability

The data used to support the findings of this study are available from the corresponding author upon request.
